# Preparation and *in vitro* Characterization of Porous Carrier–Based Glipizide Floating Microspheres for Gastric Delivery

**DOI:** 10.4103/0975-1483.80292

**Published:** 2011

**Authors:** N Pandya, M Pandya, V H Bhaskar

**Affiliations:** *Department of Pharmaceutics, M.P. Patel College of Pharmacy, Jeevanshilp Campus, Kapadwanj, Gujarat - 387 620, India*

**Keywords:** Calcium silicate, emulsion solvent diffusion method, floating controlled drug-delivery system, glipizide, microspheres

## Abstract

Floating microspheres have been utilized to obtain prolonged and uniform release of drug in the stomach for development of once-daily formulations. A controlled-release system designed to increase residence time in the stomach without contact with the mucosa was achieved through the preparation of floating microspheres by the emulsion solvent diffusion technique, using (i) calcium silicate (CS) as porous carrier; (ii) glipizide, an oral hypoglycemic agent; and (iii) Eudragit^®^ S as polymer. The effects of various formulations and process variables on the internal and external particle morphology, micromeritic properties, *in vitro* floating behavior, drug loading, and *in vitro* drug release were studied. The microspheres were found to be regular in shape and highly porous. The prepared microspheres exhibited prolonged drug release (~8 h) and remained buoyant for >10 h. The mean particle size increased and the drug release rate decreased at higher polymer concentrations. No significant effect of the stirring rate during preparation on drug release was observed. *In vitro* studies demonstrated diffusion-controlled drug release from the microspheres. Microsphere formulation CS4, containing 200 mg calcium silicate, showed the best floating ability (88% buoyancy) in simulated gastric fluid. The release pattern of glipizide in simulated gastric fluid from all floating microspheres followed the Higuchi matrix model and the Peppas-Korsmeyer model.

## INTRODUCTION

To develop oral drug-delivery systems, it is necessary to optimize both the residence time of the system within the gastrointestinal tract and the release rate of the drug from the system. Various attempts have been made to prolong the residence time of the dosage forms within the stomach.[1,2] Prolongation of the gastric residence time (GRT) of delivery devices could be achieved by promoting adhesion to the mucous membranes,[3] which acted by preventing passage of the microspheres through the pylorus[4] or by maintaining them in a buoyant fashion in gastric juice.[5–7] With regard to the floating devices, Innuccelli *et al*,[8–10] reported that an air-contained multiple-unit compartment system showed excellent buoyancy *in vitro* and prolonged GRT relative to the controls *in vivo* in the fed state. However, in the fasted state, the intragastric buoyancy of the devices did not influence GRT. Yuasa *et al*,[11] attempted to prepare an intragastric floating and sustained-release preparation, which derived its buoyancy from the air trapped in the pores of calcium silicate when these particles were covered with polymer. Murata *et al*,[12] prepared calcium-induced alginate gel beads that, upon oral administration, were capable of floating on gastric juice.

Glipizide is a second-generation sulfonylurea prescribed to treat NIDDM (non- insulin-dependent diabetes mellitus). Its short biological half-life (3.4 h) and the site of the absorption in the stomach necessitates development of controlled-release dosage forms that are retained in the stomach, which would increase the absorption, improve drug efficiency, and decrease dose requirements.[13] An objective was to develop a multiparticulate floating delivery system, consisting of highly porous carrier material like calcium silicate (CS), glipizide as the drug, and Eudragit^®^ S (ES) as the polymer, which would be capable of floating on gastric fluid and delivering the therapeutic agent over an extended period of time.

## MATERIALS AND METHODS

### Materials

Glipizide was supplied as a gift sample by Micro Labs, (Bangalore, India); CS was purchased from Sigma Chemicals (Mumbai, India); and ES (Eudragit^®^ S) was received as a gift sample from M/s Rohm Chemische GmBH (Fabrik, Germany). Ethanol, dichloromethane (DCM), and the other solvents were purchased from SD Fine Chemicals (Mumbai, India). All chemicals were of analytical-reagent grade and were used as received.

### Preparation of glipizide-absorbed CS

CS (1.0 g) was dispersed in 10 mL ethanolic solution of glipizide (50 mg) to prepare a slurry. The slurry was ultrasonicated for 10 min in an ice bath at 40% voltage frequency using a probe sonicator (Soniweld, Imeco Ultrasonics, Mumbai, India) to entrap the drug solution inside the pores of the porous carrier. The excess ethanolic solution was removed by filtration and then by drying in vacuum, which resulted in the glipizide-absorbed CS powder.[14]

### Preparation of floating microspheres

Microspheres were prepared by the emulsion solvent diffusion method established by Kawashima *et al*.[14] as follows: The glipizide-absorbed CS was added into the polymer solution of ES (1 g) in ethanol and DCM (2:1) and sonicated using the probe sonicator (Soniweld). The resulting suspension was poured into a 200 mL aqueous solution of polyvinylpyrollidine (0.75% w/v) in a 500 mL beaker at 40°C. The emulsion/suspension was stirred at 500 rpm employing a 2-bladed propeller-type agitator (Remi, Mumbai, India) for 3 h. The microspheres were separated by filtration using Whatman filter paper (No. 41, Whatman, Brentford, UK), washed with water, and dried at room temperature in a desiccator for 24 h. The microspheres of glipizide without CS (WC) were also prepared using the same method for comparative study.

### Process variables

Amount of polymer: 500, 1000, and 1500 mg; stirring rate: 250, 500, 750, and 1000 rpm; Temperature of the preparation: 20, 30, 40, and 50°C; volume of aqueous phase: 200, 300, 400, and 500 mL; solvent ratio (ethanol: DCM): 1:1, 2:1, and 3:1; amount of carrier: 50, 100, 150, 200, and 250 mg.

### Preparation of nonfloating microspheres

Nonfloating microspheres were prepared using the procedure reported by Choi *et al*.:[15] ES (1.0 g) and glipizide (50 mg) were dissolved in 10 mL of ethanol/DCM mixture (2:1), followed by addition of 1 mL of aqueous phase containing 0.25% w/v of Tween 80. The initial water/oil (w/o) emulsion was prepared by stirring the mixture for 20 s. The w/o emulsion was slowly added into 500 mL of corn oil, the second oil phase containing 0.02% w/v of Span 80 as a surfactant, with stirring at 500 rpm at 25°C. The mixture was stirred for 1 h and the hardened microspheres were collected by filtration. The collected microspheres were washed with n-hexane thrice and soaked in fresh hexane with gentle shaking for 24 h. The microspheres were separated and then dried in an oven overnight at 50°C.

### Characterization of microspheres

#### Micromeritic properties

The microspheres were characterized by their micromeritic properties, such as particle size, true density, tapped density, compressibility index, and flow properties.[16] The size was measured using an optical microscope, and the mean particle size was calculated by measuring 200–300 particles with the help of a calibrated ocular micrometer. The tapping method was used to determine the tapped density and percent compressibility index as follows:

Tapped density = mass of microspheres / volume of microspheres after tapping

$$\%compressibility\;index=\left[1–\frac{V}{V_{0}}\right]\times 100$$

Here, V and V_0_ are the volumes of the sample after and before the standard tapping, respectively. True density was determined using a benzene displacement method. Porosity (ε) was calculated using the equation:

$$\varepsilon \;=\;\left(1\;–\;P_{p}/P_{t}\right)\times 100$$

Where *P_t_* and *P_p_* are the true density and tapped density, respectively. Angle of repose Ø of the microspheres, which measures the resistance to particle flow, was determined by a fixed funnel method and calculated as

tanØ = 2H/D

Where *2H / D* is the surface area of the free standing height of the microspheres heap that is formed on a graph paper after making the microspheres flow from the glass funnel.

#### Morphology

The external and internal morphology of the microparticles and CS were studied by scanning electron microscopy (SEM). The samples for SEM were prepared by lightly sprinkling the powder on a double adhesive tape stuck to an aluminium stub. The stubs were then coated with gold to a thickness of about 300 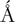 under an argon atmosphere using a gold sputter module in a high-vacuum evaporator. The coated samples were then randomly scanned and photomicrographs were taken with a scanning electron microscope (Jeol JSM-1600, Tokyo, Japan).

#### Drug content

The drug content of Eudragit^®^ S microspheres was determined by dispersing 50 mg formulation (accurately weighed) in 10 mL ethanol, followed by agitation with a magnetic stirrer for 12 h to dissolve the polymer and to extract the drug. After filtration through a 5 μm membrane filter (Millipore), the drug concentration in the ethanol phase was determined spectrophotometrically at 276 nm (Shimandzu 1601, UV-spectrophotometer). Eudragit^®^ S and the CS powder did not interfere under these conditions. Each determination was made in triplicate. The percentage drug entrapment and yield were calculated as follows:[17]

$$\%Drug\;entrapment=\left[\frac{Calculaetd\;drung\;content}{Theoretical\;drug\;content}\right]\times 100$$

$$\%Yield=\left[\frac{Total\;weight\;of\;floating\;microparticles}{Total\;weight\;of\;drug,\;polymer\;and\;porous\;carrier\;(if\;added)}\right]\times 100$$

#### Floating behavior

Fifty milligrams of the floating microparticles were placed in simulated gastric fluid (pH 2.0; 100 mL) containing 0.02% w/v Tween 20. The mixture was stirred at 100 rpm in a magnetic stirrer. After 8 h, the layer of buoyant microparticles was pipetted and separated by filtration. Particles in the sinking particulate layer were separated by filtration. Particles of both types were dried in a desiccator until a constant weight was obtained. Both the fractions of microspheres were weighed and buoyancy was determined by the weight ratio of floating particles to the sum of floating and sinking particles.[18]

$$Buoyancy\;\left(\%\right)\;=\;W_{f}\;\left(W_{f\;}+\;W_{s}\right)\times 100$$

Where *W_f_* and *W_s_* are the weights of the floating and settled microparticles, respectively. All the determinations were made in triplicate.

#### Swelling index

For estimating the swelling index, the microspheres were suspended in 5 mL of simulated gastric fluid USP (pH 1.2). The particle size was monitored by microscopy technique every 1 h using an optical microscope. The increase in particle size of the microspheres was noted for up to 8 h, and the percentage of swelling was determined at different time intervals by the difference between diameter of microspheres at time t (Dt) and initial time (t = 0 [D0]) as calculated from the following equation:

$$\mathrm{Swelling}\;\mathrm{index}=\frac{D_{t}\;–\;D_{o}}{D_{t}}$$

#### Differential scanning calorimetry

Differential scanning calorimetric (DSC) measurements were carried out on a modulated DSC (Shimadzu DSC-60 Calorimeter, Tokyo, Japan). Samples of 2–10 mg were placed in aluminium pans and sealed. The probes were heated from 25°C to 400°C at a rate of 10 K/min under nitrogen atmosphere.

#### In vitro release studies

The release rate of glipizide from floating microspheres was determined in a United States Pharmacopeia (USP) XXIII basket-type dissolution apparatus. A weighed amount of floating microspheres equivalent to 50 mg drug was filled into a hard gelatine capsule (No. 0) and placed in the basket of the dissolution rate apparatus. Five hundred millilitres of the SGF containing 0.02% w/v of Tween 20 was used as the dissolution medium. The dissolution fluid was maintained at 37°C ± 1°C at a rotation speed of 100 rpm. Perfect sink conditions prevailed during the drug release study. Five millilitre samples were withdrawn at 30 min intervals, passed through a 0.25 μm membrane filter (Millipore), and analyzed spectrophotometrically at 276 nm to determine the concentration of glipizide present in the dissolution medium. The initial volume of the dissolution fluid was maintained by adding 5 mL of fresh dissolution fluid after each withdrawal. All experiments were run in triplicate.[19]

#### Drug release pattern from microspheres

In order to understand the mechanism and kinetics of drug release, the results of the *in vitro* drug release study were fitted with various kinetic equations like zero-order (% release vs. t), first-order (log % release *vs* t), and Higuchi model (M_t_/M_∞_ *vs*. t). In order to define a model which will represent a better fit for the formulation, drug release data was further analyzed by Peppas equation, M_t_/M_∞_ = k t^n^, where M_t_ is the amount of drug released at time t and M_∞_ is the amount of drug released at time ∞; thus, the M_t_/M_∞_ is the fraction of drug released at time t, k is the kinetic constant, and n is the diffusional exponent, a measure of the primary mechanism of drug release. R^2^ values were calculated for the linear curves obtained by regression analysis of the above plots.[20,21]

## RESULT AND DISCUSSION

### Formation of microspheres

The floating microspheres were prepared by the emulsion solvent diffusion technique. A solution or suspension of Eudragit^®^ S and glipizide with CS in ethanol and dichloromethane was poured into an agitated aqueous solution of polyvinyl alcohol. The ethanol rapidly partitioned into the external aqueous phase and the polymer precipitated around dichloromethane droplets. The subsequent evaporation of the entrapped dichloromethane led to the formation of internal cavities within the microspheres. The incorporation of drug-adsorbed CS into the formulation may produce a porous structure within the microspheres. The ultrasonication produced drug-adsorbed CS in a fine state of subdivision.

A potential advantage of using large volumes of the external aqueous phase are the reduction of the required stirring times. The solubility of dichloromethane in water is 1% w/v. With larger volumes (400–500 mL), the diffusion of dichloromethane into the aqueous phase, and hence solidification of particles, occurred faster as compared to that with 200 mL. Thus, particles could be separated after shorter stirring times. It was found that a saturated solution of polymer produced smooth and high-yield microspheres. The undissolved polymer produced irregular and rod-shaped particles. Preparation at 20°C or 30°C provided porous microspheres having higher porosity, with a surface so rough as to crumble upon touching. At 40°C, polymer and the drug were co-precipitated and the shell was formed by the diffusion of ethanol into the aqueous solution and simultaneous evaporation of dichloromethane. In contrast, microspheres prepared at 50°C demonstrated a single large depression at the surface, which was a consequence of rapid evaporation of dichloromethane. A portion of the polymer solution aggregated into a fiber-like structure as it solidified prior to forming droplets or, alternatively, the transient droplets were broken before the solidification was complete. As ethanol quickly diffused out of the organic phase (polymer solution) into the aqueous phase, Eudragit^®^ S dissolved in ethanol solidified in fiber-like aggregates. It is documented that when the diffusion rate of solvent out of emulsion droplet is too slow, microspheres coalesce together. Conversely, when the diffusion rate of solvent is too fast, the solvent may diffuse into the aqueous phase before stable emulsion droplets are developed, causing the aggregation of embryonic microsphere droplets. The ratio of dichloromethane with ethanol also affected the morphology of the microspheres and the best result with a spherical shape was obtained when the ratio of ethanol to dichloromethane was 2:1. However, the average particle size increased and the wall thickness also increased as the amount of Eudragit^®^ S increased. When the amount of Eudragit^®^ S was 1.5 g in 15 mL of organic phase, it started to form aggregates. When the amount of Eudragit^®^ S was less than 0.5 g in 15 mL of organic phase, it started to form irregular microspheres with some pores. It is obvious that the rotation speed of the propeller affects the yield and size distribution of microspheres. As the rotation speed of the propeller increased from 250 rpm to 1000 rpm, the average particle size decreased, while maintaining its morphology. The optimum rotation speed for this experimental system was 500 rpm, as judged from the results of particle size and size distribution and the drug content.

### Micromeritic properties

The mean particle sizes were 143 μm for CS powder and 530, 608, 643, 725, and 835 μm for formulations containing CS in the range of 50–250mg. The particle size of the formulation WC was found to be 179 μm. The tapped density values ranged from 0.40–0.68 g/cm^3^, while their true densities ranged between 1.66–-1.95 g/cm^3^ for all the formulations, which may be due to the presence of low-density CS particles in the microspheres. The porosity of all the formulations was found to be in the range of 61%–79%. The compressibility index ranged between 26%–36%. All formulations showed excellent flowability as expressed in terms of angle of repose (<40°), except in the case of CS5, probably due to the higher content of CS. The better flow property indicates that the floating microspheres that were produced were non-aggregated [Table 1].

**Table 1:** Micromeritic properties of different floating microspheres (*n*=3)

Formulation code	Mean particle size (µm)	True density (g/cm^3^)	Tapped density (g/cm^3^)	Compressibility index (%)	Porosity (%)	Angle of repose (Ø)	
CS	143 ± 12	1.40 ± 0.10	0.21 ± 0.12	24.1 ± 0.5	87.0 ± 4	46.0 ± 5°	
WC	179 ± 19	1.66 ± 0.13	0.68 ± 0.23	25.6 ± 1.7	61.2 ± 5	30.1 ± 7°	
CS1	530 ± 20	1.77 ± 0.23	0.40 ± 0.12	26.2 ± 1.1	78.1 ± 8	32.4 ± 8°	
CS2	608 ± 16	1.73 ± 0.18	0.44 ± 0.13	27.1 ± 1.4	76.3 ± 1	34.7 ± 3°	
CS3	643 ± 19	1.83 ± 0.14	0.51 ± 0.10	29.6 ± 1.2	73.1 ± 3	36.2 ± 2°	
CS4	725 ± 18	1.88 ± 0.16	0.55 ± 0.12	33.1 ± 1.3	72.0 ± 7	38.6 ± 5°	
CS5	835 ± 21	1.95 ± 0.21	0.57 ± 0.11	35.5 ± 1.8	71.6 ± 5	42.3 ± 1°	

WC, floating microspheres of glipizide without carrier; and CS1 to CS5, floating microspheres of glipizide with calcium silicate

### Morphology

**Figure 1:**
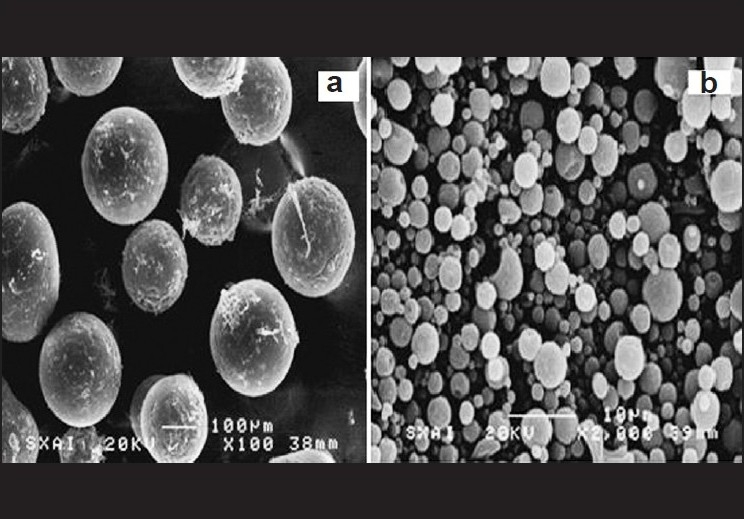
Scanning electron photomicrographs; (a) CS-based microsphere, and (b) population of microspheres. CS indicates calcium silicate

CS-based Eudragit^®^ microspheres were predominantly spherical in appearance. The porous nature of the CS and spherical shape of the microspheres are evident from their SEM photomicrographs [Figure 1a and b]. As can be seen in the photomicrograph, there are many pores and cavities in the microspheres. Low-density drug-adsorbed CS particles are clearly visible inside the microspheres, the presence of which is what make the microspheres float on the simulated GIT fluids.

### Percentage buoyancy, drug entrapment, and swelling index

The floating test was carried out to investigate the floatability of the prepared microspheres. The floating ability differed according to the formulation tested and the medium used. The microspheres were spread over the surface of SGF and the fraction of microspheres that settled down as a function of time was quantitated. All the CS-based formulations showed good floating ability (83% ± 5%). More than 80% of the particles kept floating for at least 10 h. The good buoyancy behavior of the microspheres may be attributed to the hollow nature of the microspheres and the entrapment of CS of low true density. Formulation CS4 containing 200 mg CS gave the best floating ability (88%) in SGF. Tween 20 (0.02% w/v), added to SGF, counteracted the downward pull at the liquid surface by lowering surface tension, because the relatively high surface tension of simulated gastric fluid causes the highest decrease of surface area at the air-fluid interface. Floating of microspheres for 10 h was considered satisfactory performance. It was also observed that the microspheres of larger size showed longer floating time [Table 2].

**Table 2:** Buoyancy, drug entrapment, and *in vitro* release data of different floating microspheres (*n*=3)

Formulation code	CS content (mg)	Buoyancy (%)	Drug entrapment (%)	*in vitro* drug release (8th hour) (%)	
WC	0	71	71.00	71	
CS1	50	77	78.76	59	
CS2	100	82	81.94	50	
CS3	150	84	83.87	47	
CS4	200	88	86.00	41	
CS5	250	83	80.00	33	

WC, floating microspheres of glipizide without carrier; and CS1 to CS5, floating microspheres of glipizide with calcium silicate

The percent drug entrapment of glipizide in all the formulations was found to be good (81% ± 4.0%) at all levels of drug loading. The high entrapment efficiency of glipizide is believed to be due to its poor aqueous solubility. The extent of loading influenced the particle size distribution of microspheres. When the loading was high, the proportion of larger particles formed was also high. With 80% entrapment, most of the particles were in the size range of 500–1000 μm, which is suitable for oral administration. The size of the microspheres formed may however be a function of many factors, such as stirring speed, viscosity of the dispersed phase and dispersion medium, temperature, amount and size of porous carrier, etc. Therefore, it is possible to prepare microspheres of desired size by varying some of these parameters. From the experimentally determined yields it was found that about 35% microspheres did not contain any porous carrier. The basis for this may be the difference in particle size. As porous carrier-free microspheres and carrier particles are much smaller in size (100–200 μm) than those microspheres containing carrier (500–800 μm), they were separated during the sieving step.

Similar results were obtained for swelling index. The amount of polymer directly affected the solvent transfer rate; thus, as the polymer concentration increased the swelling index also increased. The swelling index varied from 0.866–1.423. Thus, we can conclude that the amount of polymer and stirring speed directly affects swelling index.

### Differential scanning calorimetry

**Figure 2:**
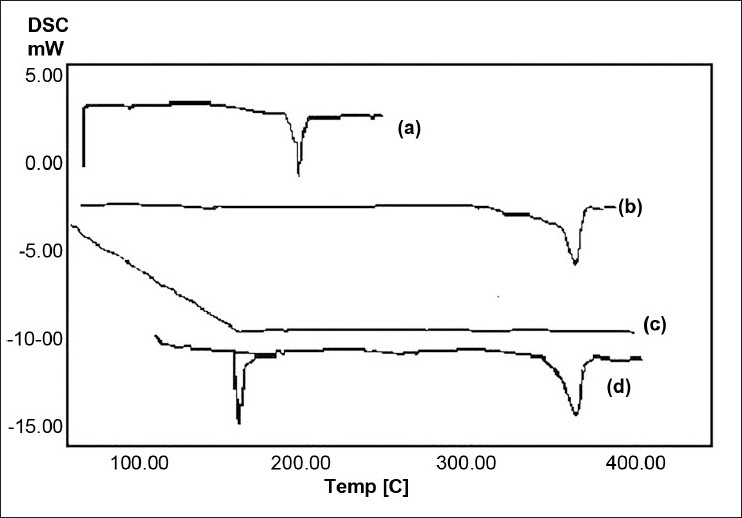
DSC thermogram of (a) glipizide; (b) CS powder; (c) Eudragit^®^ S; (d) calcium silicate–based microspheres of glipizide

In order to determine the physical state of drug, i.e., whether amorphous or crystalline, before and after floating microsphere formulation, DSC examination was conducted for the pure drug, the polymer, CS, and the optimized formulation. Thermograms of the single component(s) and microspheres are shown in Figure 2. A sharp melting transition of glipizide (pure) was observed at 207.5°C (curve A). CS powder showed a broad peak maximum at 360°C (curve B). Eudragit^®^ S showed a broad transition (curve C). A DSC thermogram of optimized formulation showed the CS peak at 360°C and one broad peak at 167°C, which might be the displaced peak of drug (curve D), suggesting that the drug is partly dissolved in the polymer and partly in the amorphous form and distributed throughout the system. Presence of the CS peak in curve D also confirms the presence of CS particles inside the formulation.

### *In vitro* drug release study

**Figure 3:**
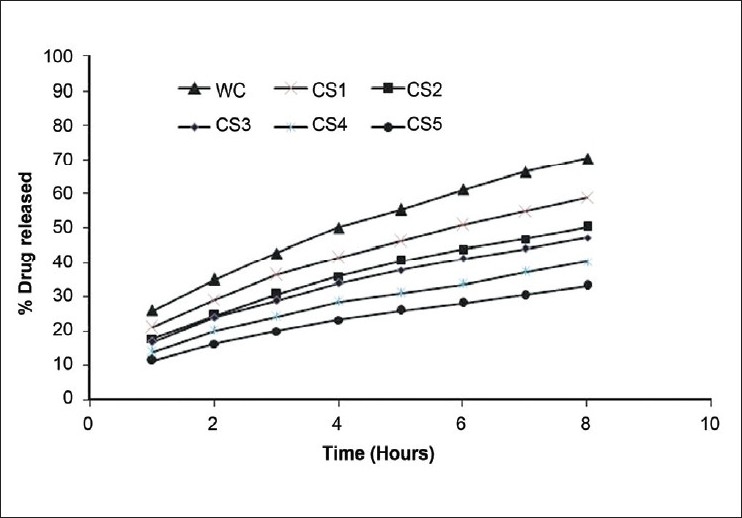
*In vitro* release of glipizide from various floating microspheres in simulated gastric fluid (pH 2.0) (*n*=3). WC indicates floating microspheres of glipizide without carrier; and CS1-5, floating microspheres of glipizide with calcium silicate

Release of glipizide from CS-based microspheres was evaluated in SGF (pH 2.0). Since the acrylic polymer used is not soluble in acidic pH and starts to dissolve only above pH 7, microspheres released the glipizide only by diffusion in SGF (pH 2.0). The other reason for the slow dissolution rate of drug may be attributed to the low solubility of glipizide at acidic pH. No burst effect was observed from any of these formulations. The release of glipizide from different formulations followed the order: WC > CS1 > CS2 > CS3 > CS4 > CS5. The pattern also provides an idea about the effect of CS content on drug release from the microspheres (i.e., the higher the CS content in microspheres, the lower the drug release) [Figure 3]. The release mechanism of glipizide from these floating microspheres was also evaluated on the basis of theoretical dissolution equations including zero-order, first-order, Higuchi matrix, and Peppas-Korsmeyer models. The regression coefficients and rate constants from *in vitro* release profiles of glipizide in SGF were calculated and are reported in Table 3. Release pattern of glipizide in SGF (pH 2.0) from all floating microspheres followed the Higuchi matrix model and the Peppas-Korsmeyer model. Desai *et al*.[5] reported that noneffervescent floating systems obeyed the Higuchi model, indicating drug release via a diffusion mechanism.

**Table 3:** The regression coefficients and rate constants for release of glipizide from floating microspheres in simulated gastric fluid (pH 2.0) (*n*=3)

Formulation	Zero-order model		First-order model		H-M model		P-K model	
code	r	K_1_	r	K_2_	r	K_3_	r	K_4_
WC	0.8720	10.684	0.9799	– 0.164	0.9902	24.671	0.9782	24.539
CS1	0.8523	8.417	0.9564	– 0.114	0.9931	19.493	0.9807	20.780
CS2	0.8652	7.307	0.9488	– 0.093	0.9953	16.907	0.9868	17.099
CS3	0.8674	6.754	0.9457	– 0.084	0.9924	15.611	0.9787	16.332
CS4	0.8629	6.158	0.9362	– 0.075	0.9898	14.235	0.9736	15.114
CS5	0.8563	5.503	0.9247	– 0.065	0.9924	12.738	0.9807	13.499

In view of the potential utility of the formulation, stability studies were carried out at 25°C/60% RH, 30°C/65% RH, and 40°C/75% RH for 6 months (climatic zone IV condition for accelerated testing) to assess their long-term (2 years) stability. The protocols of stability studies were in compliance with the guidelines in the WHO document for stability testing of products intended for the global market. After storage, the formulation was subjected to a drug assay, floating behavior, and *in vitro* dissolution studies. The analysis of the efficiency of dissolution data, floating behavior, and drug content after storage at 25°C/60% RH, 30°C/65% RH, and 40°C/75% RH for 6 months showed no significant changes in the formulations.

## CONCLUSION

The present formulation study of glipizide was performed in an attempt to prepare a floating drug delivery system consisting of a floating multiple-unit system. Incorporation of CS in the microspheres proved to be an effective method to achieve the desired release behavior and buoyancy. The performance of these formulations was evaluated and the effect of various formulation variables was studied. The designed system, combining excellent buoyant ability and suitable drug release pattern, could possibly be advantageous in terms of increased bioavailability of glipizide. The major advantages of the system include: (i) ease of preparation, (ii) good buoyancy, (iii) high encapsulation efficiency, and (iv) sustained drug release over several hours.

The developed formulation overcomes and alleviates the drawbacks and limitations of sustained-release preparations in the drug-delivery art through the introduction of CS-based floating microspheres suitable for controlled release of drug after oral administration. The microspheres could be compressed into tablets, filled into capsules, or formulated into oral suspensions for reconstitution.
